# Sarcoidosis misdiagnosed as pulmonary malignancy: a case report

**DOI:** 10.3389/fonc.2025.1722646

**Published:** 2026-01-05

**Authors:** Houlu Zhang, Yubin Huang, Xiuyi Zhao, Daming Fan, Zhongxiang Xing, Xiaoming Sun, Liangming Zhu

**Affiliations:** 1 School of Clinical Medicine, Shandong Second Medical University, Weifang, Shandong, China; 2 Department of Thoracic Surgery, Jinan Central Hospital, Jinan, Shandong, China; 3 Central Hospital Affiliated to Shandong First Medical University, Shandong First Medical University & Shandong Academy of Medical Sciences, Jinan, Shandong, China; 4 Department of Nuclear Medicine, Jinan Central Hospital, Jinan, Shandong, China; 5 Department of Pathology, Jinan Central Hospital, Jinan, Shandong, China; 6 Department of Minimally Invasive Esophageal Surgery, Tianjin Medical University Cancer Institute and Hospital, National Clinical Research Center for Cancer, Tianjin, China; 7 Key Laboratory of Cancer Prevention and Therapy, Tianjin’s Clinical Research Center for Cancer, Tianjin, China; 8 Department of Thoracic Surgery, Qilu Hospital, Shandong University, Jinan, Shandong, China

**Keywords:** sarcoidosis, intra-pulmonary metastasis, ^18^F-fluorodeoxyglucose positron emission tomography/computed tomography, case report, differential diagnosis

## Abstract

Sarcoidosis is an idiopathic multisystem disease characterized by non-caseating granulomas primarily composed of epithelioid macrophages. The lungs and lymph nodes are the most commonly affected organs. Clinical manifestations of sarcoidosis vary widely, and its radiographic features often overlap with those of malignant tumors, especially pulmonary malignancy, increasing the diagnostic challenge. This article presents a case of sarcoidosis misdiagnosed as pulmonary metastatic in a patient with a history of pulmonary malignancy. Nine months after surgery for left lung adenocarcinoma, the patient had a follow-up that revealed elevated CEA levels (14.98 ng/ml). ¹^8^F-fluorodeoxyglucose positron emission tomography/computed tomography (¹^8^F-FDG PET/CT) revealed 18F-fluorodeoxyglucose (¹^8^F-FDG) metabolic hot spots in the right upper lung lobe, bilateral pleura, and spleen, as well as metabolically active lymph nodes in several locations, including the mediastinum, hilum, hepatic hilum, omental bursa, and retroperitoneum. The largest lymph node, located in the mediastinum, had a maximum standardized uptake value (SUV max) of 21.5, indicating intense glucose metabolism and suggesting pulmonary malignancy recurrence with metastasis to the pleura and spleen. To clarify the diagnosis, we conducted a lymph node biopsy and histological examination, which ultimately confirmed sarcoidosis. This case highlights that, in patients with pulmonary malignancy, sarcoidosis can coexist with or occur alternately alongside malignancy, and the imaging features of both conditions lack specificity, potentially leading to misdiagnosis. These observations underscore that reliance on imaging alone is insufficient for differential diagnosis, and that other diagnostic methods, particularly histopathological evidence, should be integrated to achieve a comprehensive assessment.

## 1 Introduction

While the exact cause of sarcoidosis is unknown, studies suggest that it may be linked to immune system dysregulation, environmental exposure, and genetic susceptibility (1). Clinical manifestations of sarcoidosis are varied and resemble those of other diseases, especially pulmonary disorders. Imaging is essential in diagnosing sarcoidosis (2). In the lungs, sarcoidosis typically presents as bilateral hilar and mediastinal lymphadenopathy, along with multiple small pulmonary nodules that appear as high-signal lesions on ¹^8^F-FDG PET/CT (3). These features closely resemble those of pulmonary malignancy, potentially leading to diagnostic errors. This case report presents sarcoidosis misdiagnosed as pulmonary malignancy, emphasizing the importance of accurate diagnosis and treatment in clinical practice.

## 2 Case report

**Figure 1:**
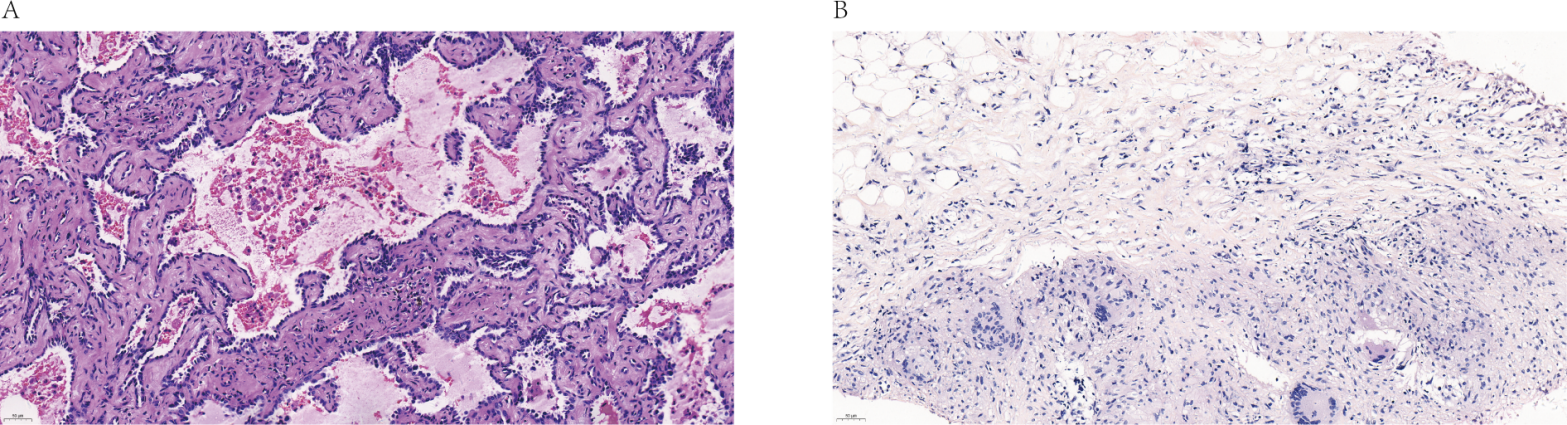
Patient’s hematoxylin and eosin (H&E)-stained image of pathological section **(A)** H&E-stained image of AIS (x400 magnification). **(B)** H&E-stained image of Sarcoidosis (x400 magnification). AIS, Adenocarcinoma in situ.

**Figure 2:**
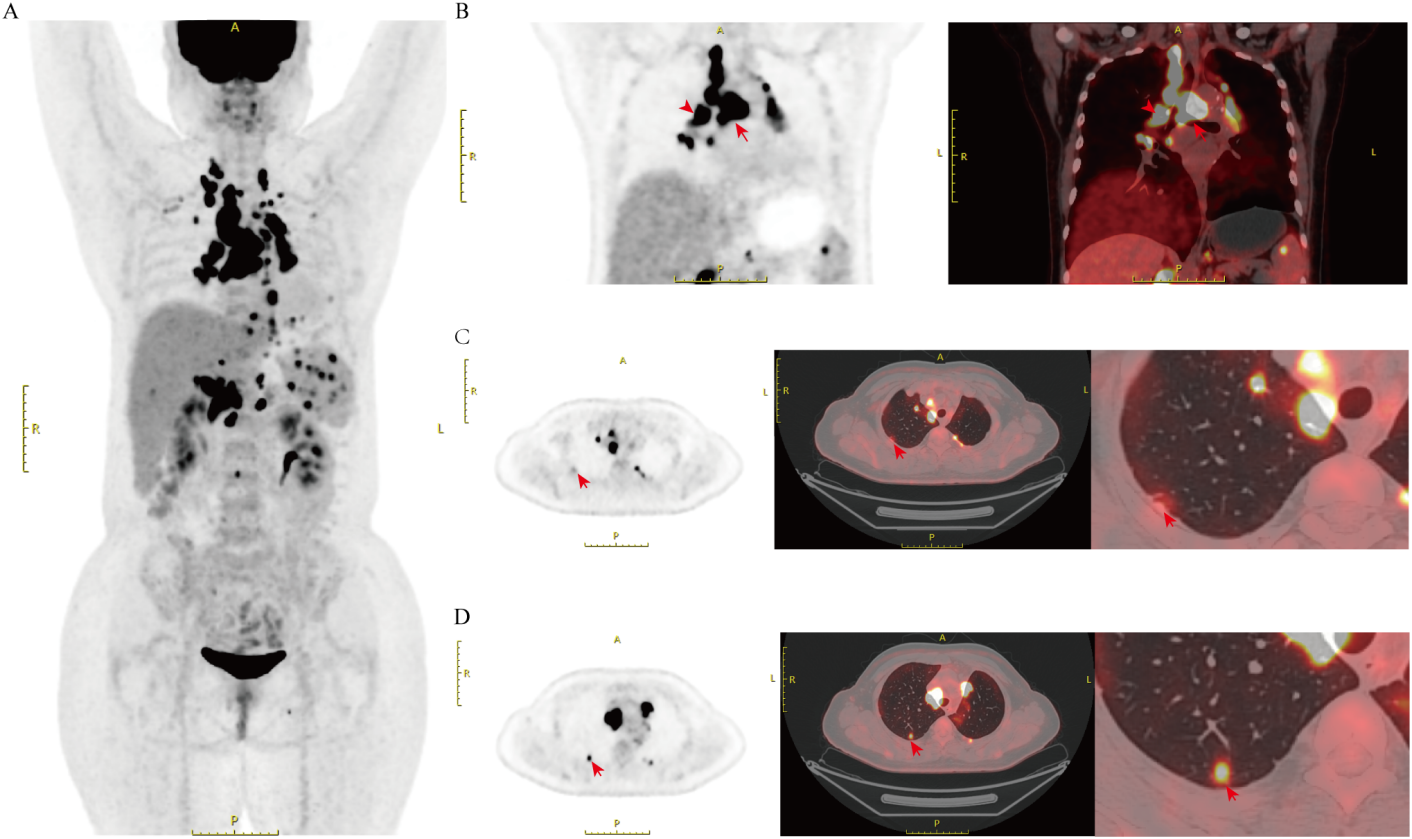
PET/CT imaging of the patient before steroid treatment **(A)** PET/CT imaging of Systemic Sarcoidosis. **(B)** PET/CT imaging of hypermetabolic lymph nodes in the mediastinum and hilum, some forming a confluent mass, with SUV max values of 17.2 and 21.5(arrows). **(C)** A markedly hyperintense lesion can be observed along the pleural surface (arrows). **(D)** Arrows point to the hypermetabolic lesions in the right upper lobe, with the SUV max of 6.8. SUV max, Standardized Uptake Value maximum.

A 53-year-old female patient presented in October 2020 with a “left upper lobe nodule” and underwent thoracoscopic left upper lobectomy and lymph node dissection. Postoperative pathology revealed a wall-adherent growth-type adenocarcinoma in the left upper lobe lingula segment (approximately 1.6×1.2 cm), with the majority being *in situ* adenocarcinoma with focal invasion. No lymph node metastasis was observed (**Figure 1A**). Based on these findings, the tumor was staged as pT1bN0M0, corresponding to pathological stage IA. Nine months after surgery, the patient’s follow-up showed elevated CEA levels (14.98 ng/ml). Chest CT showed multiple enlarged lymph nodes in the mediastinum and bilateral hila, suggestive of metastasis. Multiple pulmonary nodules were observed in both lungs, with an increase in the size of the right upper lobe nodule, suggesting potential metastasis. Based on imaging and CEA results, we suspected that the patient might have recurrent pulmonary malignancy with metastasis. To further clarify the diagnosis, we performed a ¹^8^F-FDG PET/CT scan, which showed multiple FDG-avid lymph nodes in the bilateral cervical, thoracic, abdominal, and left inguinal regions (**Figure 2A**). Focal FDG uptake was also observed in the liver and left adrenal gland, along with several FDG-avid foci of varying sizes in the spleen (SUV max=10.1). Additionally, FDG-avid lymph nodes were observed in the mediastinum, some of which formed clusters (SUV max=21.5). Similarly, FDG-avid lymph nodes were identified at both pulmonary hila (SUV max=17.2, **Figure 2B**). Bilateral pleural thickening with FDG uptake foci was observed as well (SUV max=12.1, **Figure 2C**). Moreover, an FDG-avid nodule was identified in the right upper lobe (SUV max=6.8, **Figure 2D**), raising the possibility of lung cancer recurrence with metastasis to multiple sites, including the pleura and spleen.

**Figure 3:**
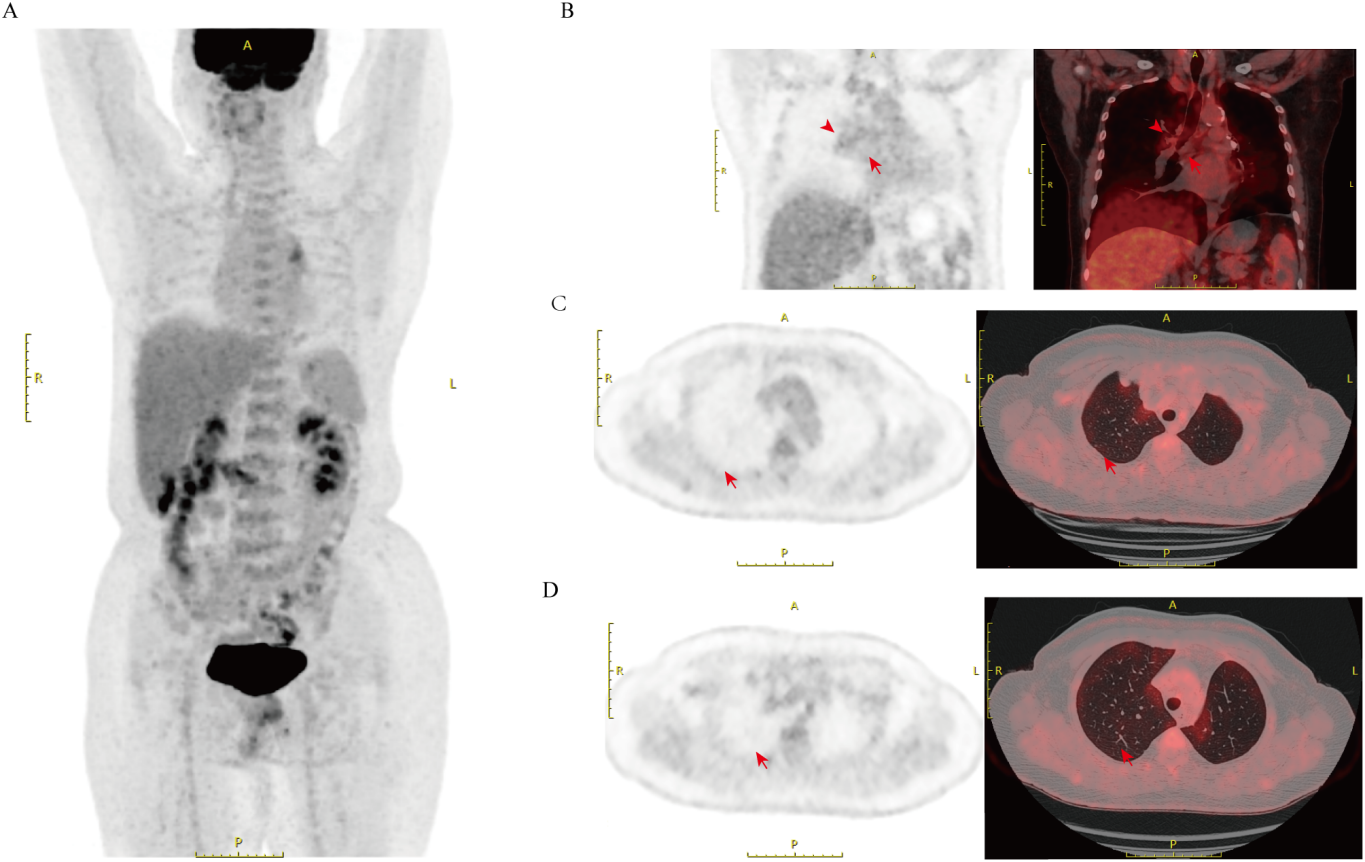
PET/CT imaging of the patient after steroid treatment **(A)** Follow-up PET/CT imaging of the same patient after treatment. **(B)** The mediastinal and hilar lymph nodes previously showing hypermetabolism are now normalized, with no high signal observed. **(C)** The previously observed hypermetabolic lesion along the pleural surface has resolved, with no visible high signal (arrows). **(D)** The hypermetabolic lesions in the right upper lobe have disappeared, with SUVmax no longer elevated (arrows).

A follow-up CT performed three months earlier revealed no significant new lesions, whereas lung malignancies typically require a longer period to involve multiple mediastinal and hilar lymph nodes. This indicates that the growth pattern of the newly detected lesions is atypical for typical malignant tumors. Consequently, we questioned the radiological diagnosis of lung cancer recurrence and metastasis. Considering that the right cervical lymph node also had a high uptake value (SUVmax=18.6), along with the safety and technical feasibility of the procedure, we chose to perform a biopsy on the right cervical lymph node instead of the mediastinal lymph node with a higher uptake value. The biopsy of the right cervical lymph node was performed, and the pathological results showed a small number of granulomatous structures with no caseous necrosis, suggesting sarcoidosis (**Figure 1B**). The clinicians also conducted a T-SPOT test, which yielded a negative result, thereby ruling out tuberculosis. Based on the comprehensive test results, the clinical diagnosis was sarcoidosis. The patient was treated with prednisone at 0.5 mg/kg (35 mg daily: 20 mg in the morning, 15 mg in the afternoon), with a gradual dose reduction after 4 weeks. After 8 months of steroid treatment, the patient underwent a follow-up ¹^8^F-FDG PET/CT scan, which showed that the previously observed FDG metabolic hot spots in the right upper lobe nodule, bilateral pleura, and multiple enlarged lymph nodes had disappeared (**Figures 3A–D**).

## 3 Discussion

Sarcoidosis can affect multiple organs, with approximately 90% of cases involving the lungs (4). Common secondary organs affected by sarcoidosis include the skin, eyes, liver, heart, and peripheral lymph nodes, with involvement rates ranging from 10% to 30% (5). About 70% of sarcoidosis patients experience spontaneous remission without treatment. If the disease remains active, pulmonary involvement may progress from asymptomatic lymphadenopathy to fibrosis and, in severe cases, respiratory failure (1).

The diagnosis of sarcoidosis relies on three primary criteria: consistent clinical manifestations, radiographic evidence, the identification of non-caseating granulomas in one or more tissue samples, and the exclusion of other granulomatous diseases (6). Commonly used imaging techniques include chest X-ray, chest computed tomography (CT), and ¹^8^F-FDG PET/CT. Chest X-ray is the standard initial screening method for sarcoidosis, typically showing bilateral hilar lymphadenopathy, small pulmonary nodules, and increased lung markings. The classic Scadding staging system differentiates the stages of disease progression based on chest X-ray findings (7). Chest CT offers higher-resolution images, allowing clearer visualization of hilar lymph nodes, small pulmonary nodules, and interstitial changes. It is advantageous in assessing the extent, distribution, and activity of lung involvement. Therefore, Chest CT not only reduces the diagnostic challenges of sarcoidosis but also aids in treatment planning and prognosis evaluation (8). ¹^8^F-FDG PET/CT is important for assessing the severity of sarcoidosis and multiorgan involvement, as well as for reflecting changes in inflammatory activity, providing a basis for treatment evaluation and adjustment (9). Additionally, it can diagnose isolated cardiac sarcoidosis even without histological evidence (10).

It is noteworthy that the similarity in the distribution and morphology of sarcoid lesions and pulmonary metastatic tumors means that even high-resolution CT (HRCT) may not reliably distinguish sarcoid nodules from other malignant or benign entities, such as sarcomatoid lesions or lymph node involvement (11). Moreover, ¹^8^F-FDG PET/CT is a critical tool for distinguishing between benign and malignant lesions, utilizing fluorine-18 labeled glucose analog FDG as a tracer. FDG is absorbed by cells via the same mechanism as glucose, but it cannot undergo dephosphorylation, preventing its entry into the tricarboxylic acid cycle (12). As a result, FDG accumulates within the cells. This leads to varying SUV values in ¹^8^F-FDG PET/CT (13). However, FDG lacks tumor specificity, meaning both tumor cells and immune cells involved in inflammatory responses may accumulate FDG due to increased glucose uptake, resulting in similar uptake values. Therefore, ¹^8^F-FDG PET/CT alone is insufficient for differentiating between the two. Several studies have suggested a potential link between malignant tumors and the onset of sarcoidosis, though the underlying mechanisms remain unclear. H. S. Pandha et al. propose that immune changes induced by malignant tumors and their treatments may trigger sarcoidosis (14). Rayson D posits that paraneoplastic syndrome could be a potential trigger for sarcoidosis development (15). Although this unexplained comorbidity increases the risk of misdiagnosis, it also guides key differential diagnosis in clinical practice.

A review of this misdiagnosis identifies several principal contributing factors. First, the patient’s history of malignant tumors restricted the clinician’s thinking, causing an overemphasis on recurrence or metastasis and neglecting other potential diagnoses. Second, sarcoidosis and malignant tumors exhibit an unclear comorbidity (16), which can cause their potential overlap, increasing diagnostic complexity. Finally, sarcoidosis and pulmonary metastatic tumors share overlapping radiographic features, with similar uptake values on ¹^8^F-FDG PET/CT, further complicating the differential diagnosis. This feature has also been noted in other case reports (17).What distinguishes the present case is that the patient’s carcinoembryonic antigen (CEA) level exceeded three times the upper limit of normal (0–5 ng/ml), accompanied by involvement of atypical sites for sarcoidosis, such as the pleura. (18). Collectively, these factors interacted to produce the observed diagnostic error.

In postoperative follow-up of pulmonary malignancy patients, accurately diagnosing the nature of new lesions prevents unnecessary treatments due to false positives and avoids delays in tumor recurrence intervention caused by false negatives, ultimately improving survival outcomes. This case highlights the inadequacy of relying solely on one diagnostic method, particularly when imaging findings are ambiguous. In such cases, adequate histopathological evidence should be utilized. Furthermore, clinicians must not be constrained by the patient’s history of malignancy. Critical thinking must be employed to ensure the optimal treatment plan for the patient.

## Data Availability

The original contributions presented in the study are included in the article/supplementary material. Further inquiries can be directed to the corresponding authors.
